# Blood–brain barrier dysfunction developed during normal aging is associated with inflammation and loss of tight junctions but not with leukocyte recruitment

**DOI:** 10.1186/s12979-015-0029-9

**Published:** 2015-03-07

**Authors:** Mina Elahy, Connie Jackaman, John CL Mamo, Virginie Lam, Satvinder S Dhaliwal, Corey Giles, Delia Nelson, Ryusuke Takechi

**Affiliations:** CHIRI Institute for Ageing and Chronic Disease, Curtin University, Bentley, 6102 WA Australia; School of Public Health, Faculty of Health Sciences, Curtin University, Bentley, 6102 WA Australia; School of Biomedical Sciences, Faculty of Health Sciences, Curtin University, Bentley, 6102 WA Australia

**Keywords:** Aging, Blood–brain barrier, Inflammation, Neurodegenerative disorder, Neuroinflammation, Leukocyte infiltration, Tight junction complex

## Abstract

**Background:**

Functional loss of blood–brain barrier (BBB) is suggested to be pivotal to pathogenesis and pathology of vascular-based neurodegenerative disorders such as Alzheimer’s disease. We recently reported in wild-type mice maintained on standard diets, progressive deterioration of capillary function with aging concomitant with heightened neuroinflammation. However, the mice used in this study were relatively young (12 months of age) and potential mechanisms for loss of capillary integrity were not investigated *per se*. The current study therefore extended the previous finding to investigate the effect of aging on BBB integrity in aged mice at 24 months and its potential underlying molecular mechanisms.

**Results:**

Immunomicroscopy analyses confirmed significantly increased capillary permeability with heightened neuroinflammation in naturally aged 24-month old mice compared to young control at 3 months of age. Aged mice showed significant attenuation in the expression of BBB tight junction proteins, occludin-1 and to lesser extent ZO-1 compared to young mice. In addition, TNF-α in cerebral endothelial cells of aged mice was significantly elevated compared to controls and this was associated with heightened peripheral inflammation. The expression of ICAM-1 and VCAM-1 remained unelevated, and no sign of leukocyte recruitment was observed in aged mice.

**Conclusion:**

The BBB breakdown that occurs during ordinary aging is associated with inflammation and disruption of tight junction complex assembly but not through leukocyte trafficking.

**Electronic supplementary material:**

The online version of this article (doi:10.1186/s12979-015-0029-9) contains supplementary material, which is available to authorized users.

## Background

The blood–brain barrier (BBB) is characterized with tightly opposed endothelial cells and underlying basement membranes to separate the central nervous system from peripheral circulation. An accumulating body of evidence suggests that disruption of BBB function followed by blood-to-brain extravasation of circulating neuroinflammatory molecules may increase risk for the onset and progress of cerebrovascular-based neurodegenerative disorders such as Alzheimer’s disease (AD), vascular dementia (VaD) and multiple sclerosis [1,2]. Consistent evidence is provided in clinical and animal model studies where cerebral extravasation of circulating proteins, perivascular gliosis and lacunar lesions are commonly reported [1,3].

Aging is the most significant risk factor for vascular-based neurodegenerative disorders, and in the US more than 50% of population aged 80 years or older presents with AD or VaD. Several studies have now shown that the function and structure of BBB deteriorate during aging and in the absence of comorbidities [4,5]. The cerebrospinal fluid/serum ratio of albumin, a surrogate marker of increased capillary permeability is significantly elevated with aging [6,7]. In addition, recent studies suggest that increased BBB permeability in aged rodent brains is associated with reduced expression of BBB tight junction proteins [8,9]. Our previous study showed that in mid-aged 12 months old wild-type C57BL/6 J mice, increased cerebrocapillary permeability was shown to be associated with heightened neuroinflammation, however mechanisms involved in BBB disruption were not explored [10].

Disrupted tight junctional complex assemblies and active leukocyte trafficking may be central to cerebral capillary function. Assembly of BBB junctional complexes may be regulated by inflammatory pathways. *In vitro* and *in vivo* studies demonstrated that pro-inflammatory cytokines including TNF-α and IL-1β regulates the expression of tight junction proteins occludin-1, claudin-5, ZO-1 and ZO-2 [11-15]. Leukocyte trafficking and recruitment is a process that describes the paracellular transmigration of circulating leukocytes through the vascular wall into perivascular space of the brain [16]. Increased expression of adhesion molecules such as vascular cell adhesion molecules (VCAM-1) and intercellular adhesion molecules (ICAM-1) are reported to initiate this process by promoting leukocyte rolling and firm adhesion to BBB endothelial cell surface [17,18]. Studies also demonstrate that TNF-α upregulates the expression of ICAM-1 and VCAM-1 [19]. However, involvement of these pathways in aging induced BBB dysfunction has not been reported in naturally aged wild-type mice.

This study explored capillary integrity, inflammation and the expression of proteins central to leukocyte recruitment in very aged 24-month old C57BL/6 J.

## Results

**Figure 1:**
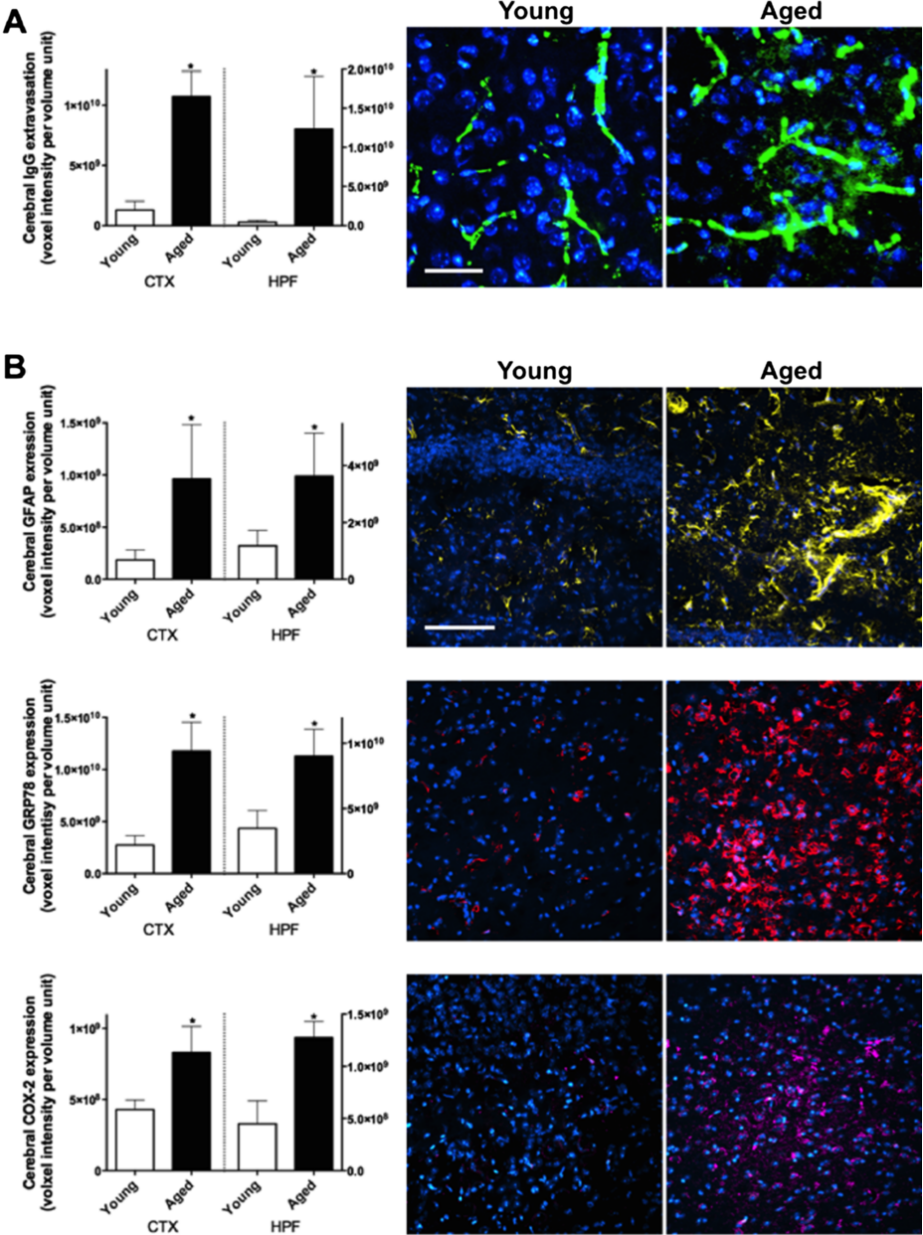
**Blood–brain barrier integrity and neuroinflammation. (A)** The integrity of BBB was assessed by measuring the blood-to-brain extravasation of IgG with semi-quantitative confocal microscopy in the cortex (CTX) and hippocampal formation (HPF) of 3 months old young mice and 24 months old aged mice. The voxel intensity of protein of interest is expressed as per volume unit. Asterisks indicate statistical significance assessed with two-tailed *t*-test (*p* < 0.05, n = 6). Representative immunomicrographs are also shown (green = IgG, blue = DAPI, scale bar = 20 μm). **(B)** The astroglial activation, neuronal ER stress and inflammation were assessed by measuring the expression of GFAP, GRP78 and COX-2 respectively. Representative immunomicrographs are shown (yellow = GFAP, red = GRP78, magenta = COX-2, blue = DAPI, scale bar = 50 μm).

The mice aged to 24 months were otherwise healthy and had no adverse event recorded. The extravasation of circulating IgG into cerebral parenchyme was significantly increased in the cortex and hippocampal formation of aged mice compared to young control mice (Figure 1A). Significantly elevated neurovascular inflammation and neuronal stress were also indicated with increased expression of GFAP, GRP78 and COX-2 in 24 months old mice (Figure 1B).

**Figure 2:**
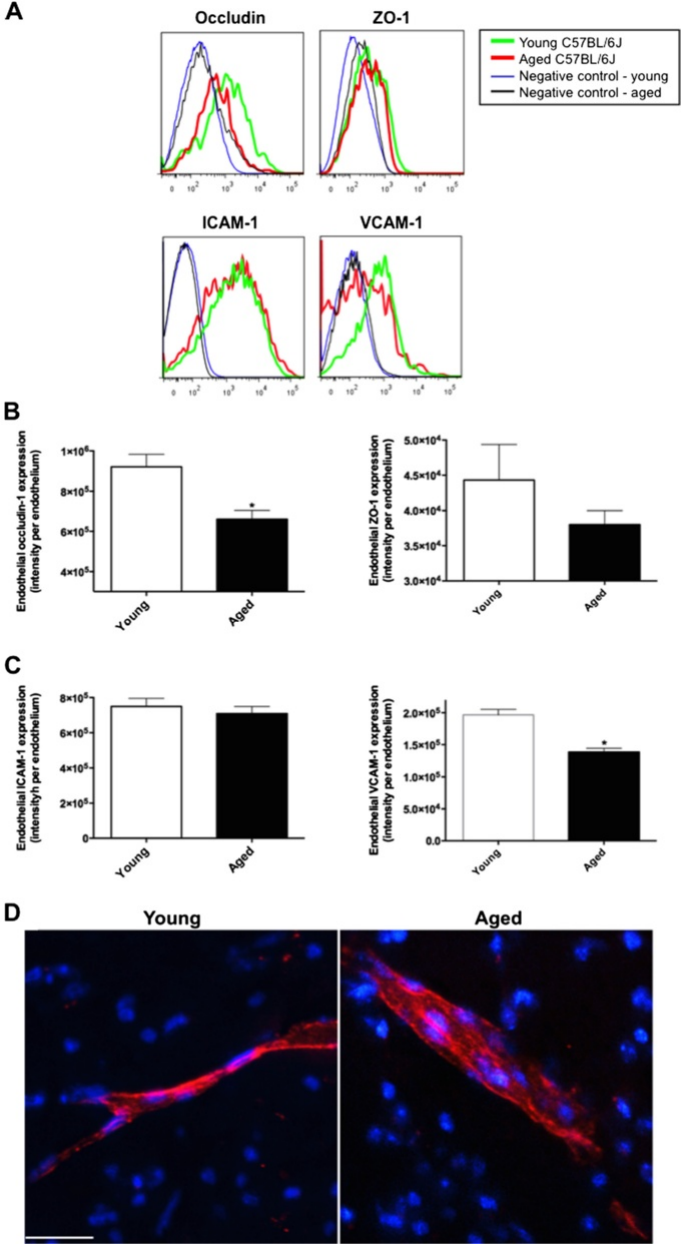
**Cerebrovascular tight junction proteins and leukocyte recruitment.** BBB tight junction assembly was assessed by measuring endothelial cell expressions of occludin-1 and ZO-1 with flow cytometry in the brains of 3 months old young mice and 24 months old aged mice. Example plots are in **(A)** and the fluorescent intensity of protein of interest is expressed as per endothelial cell **(B)**. Asterisks indicate statistical significance assessed with two-tailed *t*-test (*p* < 0.05, n = 6). **(C)** Endothelial expression of adhesion proteins, ICAM-1 and VCAM-1, was measured with flow cytometry. **(D)** The infiltration of leukocytes across the BBB was assessed with immunomicroscopy staining of CD45 immunoreactivity within the perivascular region of entire cortex and hippocampal formation. Representative images are shown. Scale bar indicates 20 μm.

The analyses with flow cytometry showed that expression of BBB tight junction protein occludin-1 was significantly attenuated in aged mice compared to control, while the expression of ZO-1 was modestly decreased in aged rats (Figure 2A, B). No significant increase in endothelial expression of ICAM-1 was observed and VCAM-1 was decreased in aged mice compared to young control (Figure 2C). Perivascular immunomicroscopy analysis showed no sign of circulating leukocyte transmigration in entire cortex and hippocampal formation of aged mice, and representative images are shown in Figure 2D. Flow cytometry of the whole brain also showed that less than 1% of total cells were CD45 positive regardless of age, and no significant difference between young vs aged mice was observed (data not shown).

**Figure 3:**
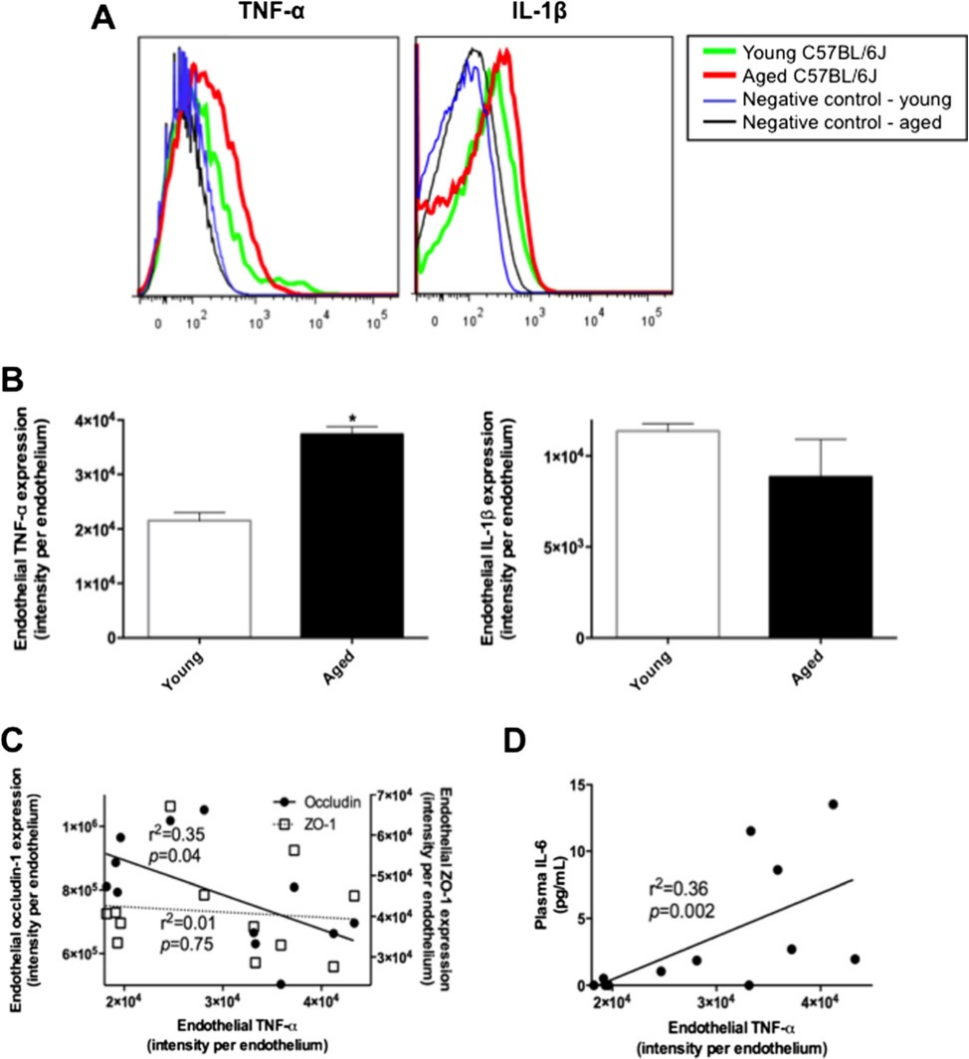
**Blood–brain barrier endothelial levels of pro-inflammatory cytokines.** The intracellular levels of TNF-α and IL-1β in cerebrovascular endothelial cells were measured with flow cytometry in the brains of 3 months old young mice and 24 months old aged mice. Example plots in **(A)** and mean of n = 6 in **(B)**. Asterisks indicate statistical significance assessed with two-tailed *t*-test (*p* < 0.05, n = 6). **(C)** The causal association of attenuated BBB tight junction proteins, occludin-1 or ZO-1, with endothelial TNF-α was analyzed with Pearson’s correlation coefficient (n = 12). **(D)** The association between the cerebrovascular TNF-α and peripheral IL-6 was determined with Pearson’s correlation coefficient (n = 12).

The BBB endothelial concentration of TNF-α was significantly increased in aged mice compared to young mice, whereas no change was seen in the intracellular level of IL-1β (Figure 3A, B). Pearson’s correlation coefficient analysis showed that endothelial levels of TNF-α were negatively correlated with BBB occludin-1 expression (*p* = 0.04), whilst a weaker negative correlation was found with ZO-1 (*p* = 0.75) (Figure 3C). The cytometric bead array showed significantly heightened IL-6 in aged mice (6.38 ± 2.28 pg/mL) compared to young control mice (0.57 ± 0.31 pg/mL), whereas the levels of other pro-inflammatory cytokines, IL-10, IL-12, TNF-α and IFN-γ, remained comparable between young control and aged mice (data not shown). Pearson’s correlation analysis showed positive correlation coefficient between the peripheral IL-6 and endothelial TNF-α (*p* = 0.002) (Figure 3D).

## Discussion

A functional consequence of increased cerebral capillary permeability with aging is enhanced blood-to-brain delivery of circulating neuroinflammatory molecules. Disturbed BBB has been reported in mid-aged rodent models independent of co-morbidities or the provision of pro-inflammatory diets. The mechanisms for the aging-induced effects on capillary function are not yet delineated. The present study extended previous studies and investigated in very aged mice, whether inflammation and/or leukocyte recruitment are associated with loss of tight junction proteins.

Consistent with previous findings, semi-quantitative immunomicroscopy analyses confirmed aging-induced BBB dysfunction with substantially increased parenchymal abundance of IgG in 24 months old wild-type mice compared to 3 months old young control. Increased cerebral expression of GFAP showed significantly increased astrocytosis and astrogliosis in the cerebral perivascular parenchyme and cerebrovascular astrocytic end feet of naturally aged mice compared to young control. In addition, significantly elevated immunoreactivity of GRP78 and COX-2 mainly in the perinuclear region of neuronal cells demonstrated substantial cellular ER stress and inflammation in the aged mice. The findings are consistent with the notion that BBB dysfunction with aging may increase risk for vascular-based neurodegenerative disorders.

Only several studies have investigated potential mechanisms involved in BBB breakdown with normal aging and these suggest heightened inflammatory processes [20]. *In vitro* and *in vivo* studies show that TNF-α potentiates the permeability of BBB by suppressing the expression of tight junction complexes [11,12,21], whilst inhibition of TNF-α, or treatment with anti-results in restoration of the tight junction protein expression and normalized BBB integrity [13]. Similarly, anti-TNF-α antibodies were shown to attenuate BBB permeability via restored expression of BBB tight junction proteins in rat model of acute liver failure [22]. In this study, exaggerated endothelial TNF-α in aged mice was associated with reduced expression of the BBB tight junction proteins, occludin-1 and ZO-1. In addition to the effects on tight junction protein expression, Previous *in vitro* studies showed TNF-α significantly increases the expression of BBB endothelial ICAM-1 and VCAM-1, which can facilitate the adhesion and transmigration of leukocytes across BBB [18]. In the very aged mice studied, there was no evidence of increased adhesion molecule expressions and leukocyte infiltration across BBB. Supporting data is presented by Miguel-Hidalgo *et al.* showing that in human 60–86 years old orbitofrontal cortex, the cerebrovascular expression of ICAM-1 remained unchanged compared to younger brain (27–54 years old) [23].

A number of studies reported that chronically elevated pro-inflammatory cytokines including TNF-α, IL-1β and IL-6 in the periphery occurs with normal aging [24,25]. When BBB endothelial cells are exposed to chronically heightened peripheral circulating inflammatory cytokines, expression of NF-κB subunits becomes significantly exaggerated [12]. NF-κB is one of the major transcription factors for inflammatory responses and triggers the secretion of pro-inflammatory cytokines including TNF-α and IL-1β. Elevated NF-κB expression and activity are also reported to promote cerebrovascular endothelial leukocyte infiltration by up-regulating expression of adhesion molecules through elevated inflammatory cytokines [26-28]. Similarly, augmentation of NF-κB subunits is involved in the suppression of tight junction proteins including occludin-1, claudin-5, ZO-1 and JAM-1 of BBB through increased pro-inflammatory cytokines [29]. IL-1β can induce the degradation of tight junction proteins including claudin-5, ZO-1 and ZO-2 [14,15,30] and treatment with anti-IL-1β antibody suppressed the exaggerated BBB permeability of *in vitro* endothelial monolayer model exposed to hypoxia [31,32]. Consistent with the potential detrimental effects of increased vascular exposure with aging to cytokines on capillary function, the concentration of IL-6 in the peripheral circulation was significantly increased and positively correlated with the cerebrovascular endothelial TNF-α in 24-month old wild type mice of present study.

Collectively, the findings of this study suggest that the mechanisms of BBB dysfunction that occurs in normal aging may result from the loss of endothelial tight junctions, induced by pro-inflammatory TNF-α through heightened peripheral inflammation, but not from leukocyte recruitment mediated by endothelial adhesion molecules. BBB leukocyte infiltration may more likely be involved in the pathological state of BBB dysfunction. The outcomes of this study offer an insight into the mechanisms involved in capillary dysfunction with normal aging.

In the present study, the ageing effect was only considered in female mice. Studies report that male mice are more vulnerable to aging related changes of capillary integrity, possibly because of andogenic hormone effects [33]. Further studies to investigate potential gender differences are appropriate. Nonetheless, the broader findings of this study suggest that disturbance in capillary function may be causally associated with neuroinflammation in aging.

## Materials and methods

### Animals

Six female wild-type C57BL/6 J mice were purchased from Animal Resources Centre (WA, Australia). Mice were maintained at Curtin University Animal Facility until 24 months of age with 12 h light/dark cycle, *ad libitum* access to standard chow and water. Only healthy, disease free mice were used in this study. As a young control group, 6 mice at age of 3 months were also purchased from Animal Resources Centre at the time of sacrifice. All mice were anesthetized with isoflorane and killed with cervical dislocation. Plasma samples were collected and stored at −80°C. The right hemisphere of the brain tissue was carefully removed, fixed in 4% paraformaldehyde for 24 h, and frozen in isopentane/dry ice for immunohistochemistry staining. The left brain hemisphere was collected in FACs buffer (2% FBS, 1% BSA in PBS) with 10 μg/ml brefeldin (Sigma-Aldrich) for flow cytometry analysis. All animal procedures described in this study were approved by a National Health and Medical Research Council of Australia approved Animal Ethics Committee (Curtin University, approval no. AEC_2012_21).

### Three-dimensional semi-quantitative immunomicroscopy for parenchymal IgG extravasation

The BBB integrity was considered by measuring the parenchymal abundance of, IgG using semi-quantitative confocal immunomicroscopy as described previously [34-36]. Briefly, after blocking with 10% goat serum, 20 μm cryosections were incubated with goat anti-mouse IgG conjugated with Alexa488 (1:50, LifeTechnologies) for 20 h at 4°C. The sections were counterstained with DAPI. A minimum of eight and five 3-D images were captured randomly from the cortex and hippocampal formation region of the brain section, respectively with UltraVIEW Vox spinning disc confocal microscope (PerkinElmer). Total image area captured and quantified represented approximately 60% of the hippocampal formation and cortex. The voxel intensity of fluorescence of each 3-D image was analyzed with Volocity imaging software (PerkinElmer), and averaged within each region by using all eight or five 3-D images to estimate the representative voxel intensity per region per mouse. Then the mean voxel intensity of IgG extravasation was calculated within each treatment group (n = 6). The parenchymal staining of IgG was specifically selected and staining within the blood vessels were excluded based on pre-set threshold parameters of Volocity and thereafter confirmed for each image to ensure proper selection by identifying the nucleus of BBB endothelial cells.

### Three-dimensional semi-quantitative immunomicroscopy for neuroinflammatory and stress markers and leukocyte infiltration

The markers of neuroinflammation and neuronal stress, glial fibrillary acidic protein (GFAP), cyclooxygenase-2 (COX-2) and 78 kDa glucose-regulated protein (GRP78) were measured with immunomicroscopy as described previously [10,37,38]. Briefly, 20 μm cryosections were incubated with either rabbit anti-mouse GFAP, GRP78, or COX-2 (1:200, 1:1000 or 1:200, respectively, Abcam) for 20 h at 4°C. The sections were then incubated with goat anti-rabbit IgG conjugated with Alexa488 (1:200, LifeTechnologies) for 2 h at 20°C. Nuclei were counterstained with DAPI and the fluorescent staining was observed with UltraVIEW Vox microscope. Similar to IgG extravasation, voxel intensity of the protein of interest was determined with Volocity from at least eight and five randomly captured 3-D images in the cortex and hippocampus, respectively.

The BBB infiltration of leukocyte was assessed with immunomicroscopy detection of anti-CD45 immunoreactivity within the perivascular region of entire cortex and hippocampus for all mice. 20 μm cryosections were incubated with anti-CD45 conjugated with PerCP-Cy5.5 for 20 h at 4°C, and the fluorescent staining was observed with UltraVIEW Vox microscope.

### Flow cytometry analysis of endothelial tight junction protein, inflammatory cytokine and adhesion molecules, and cerebral leukocytes

The expression of cerebrovascular endothelial tight junction occludin-1 and ZO-1, pro-inflammatory TNF-α and IL-1β, and adhesion VCAM-1 and ICAM-1 was measured by flow cytometry as established previously with some modifications [39-41]. The left hemisphere of the brain was sliced into 0.5 mm^3^ fractions in FACs buffer with 10 μg/ml brefeldin. The tissue was then digested with 1 mg/ml collagenase IV (Sigma-Aldrich), 1 mg/ml dispase (Sigma-Aldrich), and 1 mg/ml DNase (Sigma-Aldrich) in FACs buffer with 2.5 μg/ml brefeldin at 37°C for 30 min. Samples were then triturated to obtain a single cell suspension and the cells were incubated with FACs buffer containing 20% FBS and 2.5 μg/ml brefeldin for 10 min at 4°C.

Cell suspensions were incubated with fluorescently labeled antibodies against extracellular markers: anti-CD45-PerCP-Cy5.5 (1:500, Biolegend, cat#: 103132), anti-CD31-BV421 (1:200, Biolegend, cat#: 102423), anti-VCAM-1-APC (1:200, Biolegend, cat#: 105718), and anti-ICAM-1-PE (1:200, Biolegend, cat#: 116108). For intracellular staining, cells were fixed in 1% paraformaldehyde (Sigma-Aldrich) and permeabilised with 0.1% saponin (Sigma-Aldrich) for 15 min at 4°C. The cells were then incubated with antibodies against intracellular markers: rabbit anti-occludin-1 unconjugated (1:50, Abcam, cat #:ab167161), anti-TNF-α-APC (1:50, Biolegend, cat: 506308), anti-IL-1β-PE (1:50, eBioscience, cat#: 12-7114-82) or rabbit anti-ZO-1 unconjugated (1:100, Abcam, cat #:ab59720) for 30 min at 4°C. Subsequently the cells were incubated with anti-rabbit IgG Alexa488 (1:500) for 30 min at 4°C, and washed with FACs buffer.

Samples were acquired on a FACS Canto II (BD Biosciences). Cells were first gated so that only viable, single cells were analyzed and cerebrovascular endothelial cells were then identified as CD31 positive CD45 negative. To validate the staining and account for any potential differences between the young and aged endothelial cells, including autofluorescence, a combination of isotype controls and other internal staining controls were used (example gating shown in Additional file 1: Figure S1). The fluorescent intensity of occludin-1, ZO-1, TNF-α, IL-1β, VCAM-1 and ICAM-1 was analyzed with FlowJo V10 software (Treestar) and expressed per endothelial cell.

### Plasma levels of inflammatory cytokines

Plasma concentrations of IL-6, IL-10, IL-12, TNF-α, IFN-c and MCP-1 were determined using the mouse inflammation cytokine bead assay (BD Biosciences) according to the manufacturer’s instruction. Briefly, samples (diluted 1 in 2 in assay buffer) and standards were incubated with fluorescently labeled capture and detection beads for 2 hrs. Following washing, samples and standards were acquired on a FACS Canto II. Analysis was performed using FlowJo V10 software and sample concentration calculated from the standard curve generated for each cytokine using GraphPad Prism.

### Statistical analysis

Each young control and aged group had 6 mice to provide sufficient power to compare the effect of Aging on BBB function and structure which was determined based on previous studies where BBB function was analysed with same or similar methods and models [35,42,43]. The present study was analysed using Mice similar to “blocks” within the student *t*-test. Hence individual measurements have been adjusted for the Mice or “block” effect, which was essentially used as a covariate. Student *t*-test was used to determine the statistical significance between control and aged group (*p* < 0.05). Pearson’s correlation coefficient analysis was used with pooled data of both control and aged group to determine significant associations (n = 12, *p* < 0.05).

## Additional file

Additional file 1: Figure S1. Gating strategy for brain endothelial cells. Brains from young (2 months) or aged (24 months) C57BL/6 J mice were collected for flow cytometric analysis. After gating on single cells, endothelial cells were identified as CD45^neg^CD31^+^ cells. Boundaries for the gating selection were determined using fluorescence minus one controls and representative plots are shown.
