# Supplementary figures and images for: Blood–brain barrier dysfunction developed during normal aging is associated with inflammation and loss of tight junctions but not with leukocyte recruitment
Source: Immun Ageing. 2015 Mar 7;12:2. doi: 10.1186/s12979-015-0029-9 (PMC4362825; doi:10.1186/s12979-015-0029-9)

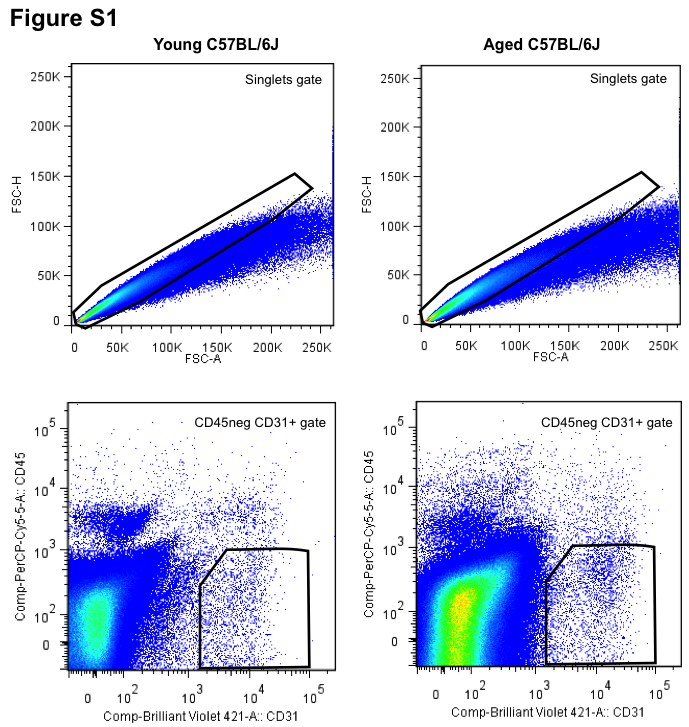

Supplement: Additional file 1: Figure S1. — Gating strategy for brain endothelial cells. Brains from young (2 months) or aged (24 months) C57BL/6 J mice were collected for flow cytometric analysis. After gating on single cells, endothelial cells were identified as CD45negCD31+ cells. Boundaries for the gating selection were determined using fluorescence minus one controls and representative plots are shown. [file 12979_2015_29_MOESM1_ESM.tiff]
